# Direct Acoustic Versus AUV-Assisted Hybrid Reporting for Subsea Pipeline Monitoring: A Matched End-to-End Evaluation

**DOI:** 10.3390/s26196337

**Published:** 2026-10-08

**Authors:** Aissata Mamadou Diallo, Najat Rafalia, Jaafar Abouchabaka

**Affiliations:** Department of Computer Science, Faculty of Sciences, Ibn Tofail University, Kenitra 14000, Morocco

**Keywords:** subsea pipeline monitoring, AUV data collection, hybrid acoustic and optical communication, deadline-aware architecture, end-to-end delivery, energy accounting

## Abstract

Subsea pipeline monitoring must deliver urgent alarms quickly while moving much larger inspection records over constrained underwater links. This creates a deployment choice: send all data through a prioritized acoustic network, or collect routine records with an autonomous underwater vehicle (AUV) undertaking a finite collection mission. We compare these complete reporting architectures under identical traffic, geometry, deadlines, available retry choices, and surface infrastructure. A predefined parameter search, independent confirmation and deployment controls test complete measurement recovery under finite storage, communication and vehicle resources. In the reference deployment, direct priority recovered **100%** of deadline-eligible raw measurement windows on time, compared with **0.84%** for the selected hybrid deployment. Lower submerged-equipment energy in the hybrid case coincided with extensive retained data and greater total mission energy. The result applies to the tested mission and controllers. It identifies collection opportunities, final transfer and mission availability as joint constraints that must be satisfied before local energy reductions can support a deployment decision.

## 1. Introduction

Subsea pipeline monitoring produces two communication demands with different operational consequences. Pressure transients and acoustic-emission systems can generate compact alarms that must reach a surface team quickly enough to support intervention, whereas in-line and local inspection systems produce much larger records for condition assessment and maintenance planning [1,2,3]. Both streams depend on underwater links with limited capacity, long propagation times, and battery-powered equipment that is costly to access. A deployment must therefore protect urgent reporting without making routine inspection data unnecessarily expensive to move.

Two complete service chains address that problem. A direct service keeps every record on the acoustic path and uses priority scheduling to protect alarms. A hybrid service keeps the same acoustic access for urgent traffic but assigns routine records to short optical contacts with an autonomous underwater vehicle (AUV), which carries them until surface transfer. Acoustic links provide the required reach but at a comparatively low rate, whereas optical links provide high-rate service only at close range [4,5,6]. The operational decision is whether adding AUV pickup creates enough value in alarm response, acoustic demand, and submerged energy to justify the associated waiting, incomplete service, and vehicle cost.

Prior research on pipeline networks has established linear sensor deployments, AUV collection, priority-aware mobile sinks, and resurfacing or terminal-offload decisions [7,8,9,10,11]. Optimization and learning methods then select routes, visits, links, or schedules within an AUV-assisted system [12,13,14,15,16]. These studies establish the components required for mobile collection but leave the preceding deployment question open: how does a complete hybrid service compare with a correctly prioritized direct service when demand, geometry, deadlines and surface infrastructure are shared, and both services are tuned under the same protocol?

This study answers that question through a matched end-to-end evaluation from measurement acquisition to complete surface delivery. Direct priority, static hybrid priority and a report-driven adaptive hybrid policy are tuned over declared parameter sets before independent evaluation. Finite archives, cabled ingress, acknowledged transfers and vehicle resources retain the cost and waiting time of every service stage. Instruments produce continuously over a common observation period, while hybrid collection receives one finite vehicle mission. Energy is reported for inaccessible submerged equipment and for the complete deployed system, including surface equipment and the vehicle.

This design yields three contributions:

- A matched, independently evaluated comparison of tuned direct and hybrid services, with an explicit feasibility test for adaptive collection;
- An end-to-end assessment that measures timely complete observations and locates retained data along the acquisition-to-surface service chain;
- A deployment-aware energy assessment that separates local equipment draw from total system cost and relates both to the information delivered.

The evaluation follows three questions. **RQ1** asks which tuned service satisfies the reporting requirements under finite resources. **RQ2** asks how link quality, fallback timing, deployment geometry and offered load shift that trade-off and where the active service constraint appears. **RQ3** asks under which energy boundary hybrid collection produces a deployment benefit. Section 2 positions these questions against the literature. Section 3 and Section 4 define the alternatives and evaluation method, Section 5 presents the evidence, and Section 6 translates it into a deployment decision.

## 2. Related Work and Positioning

### 2.1. Subsea Pipeline Monitoring and Mobile Collection

Pipeline-monitoring research connects integrity sensing, linear-network communication, and mobile inspection. Pressure-transient, acoustic-emission, and in-line inspection studies establish the complementary information needed for leak detection and structural assessment [1,2,3]. At the network level, Jawhar et al. [7] described an AUV-assisted pipeline architecture, Ahmed et al. [9] evaluated collection in a linear sensor network, and Goyal et al. [10] used mobile sinks for priority-based leak-monitoring traffic. Zheng et al. [8] extended the service endpoint to the surface by jointly considering the AUV tour and resurfacing decision. Across these works, topology, collection points, and the endpoint that defines delivery are central architectural choices.

Mobile service introduces waiting and vehicle motion in exchange for reduced underwater transmission. Janardanan Kartha and Jacob [17] evaluated delay, delivery, energy, and network lifetime for mobility-assisted acoustic collection, while Hollinger et al. [18] connected robotic data collection with measured channel structure and medium access. Queueing and information-value studies then separated eventual delivery from freshness and revisit time [12,19,20,21,22]. Operational studies broaden the boundary further: resident-AUV routing includes docking and battery constraints [23], and terminal-offload planning retains a final service phase until data recovery or budget exhaustion [11]. This broader view motivates evaluating the complete record journey from acquisition to surface and accounting for the vehicle mission together with fixed-node communication.

### 2.2. Priority-Aware and Hybrid Underwater Service

Acoustic and optical links occupy complementary operating regimes. Acoustics provide long reach at a comparatively low rate; optics provide high-rate local service but require close contact and suitable alignment. Event simulation, hybrid routing, and multimodal collection studies have combined these regimes for reliability, delay, and energy management [6,24,25,26,27,28]. Weng et al. [29] demonstrated three-dimensional optical tracking and seafloor-platform data retrieval in sea trials, establishing the physical plausibility of local AUV pickup. A complete hybrid service must additionally account for queue residence, vehicle carriage, and the final transfer to a surface endpoint.

Recent optimization and learning methods determine how such an AUV-assisted service should operate. Liu et al. [30] combine urgent forwarding with value-aware retrieval; Bu et al. [13] jointly select an acoustic or optical mode and an AUV trajectory; and Song et al. [14], Cao et al. [15], and Wei et al. [16] optimize motion, channel-aware collection, information freshness, or energy. Lee et al. [31] extend scheduling to the buoy endpoint through inter-AUV relay service. These methods optimize control decisions within an AUV-assisted architecture.

Two recent studies highlight additional operational dependencies. He et al. [32] study joint secure resource allocation and trajectory planning in a low-altitude aerial network, illustrating the coupling between platform motion and communication decisions. Kang et al. [33] address asynchronous-clock calibration and localization in underwater acoustic sensor networks. These studies motivate explicit motion, timing and localization assumptions; the hardware scales and simulation parameters used here are specified separately.

### 2.3. From Within-Architecture Optimization to Architecture Selection

The sensing, routing, optical-contact, scheduling, and trajectory components required for mobile collection are well established. The remaining deployment question is comparative: what operational value does the complete hybrid chain create relative to a priority-based direct chain under the same demand, geometry, deadlines, tuning protocol, and surface infrastructure? Table 1 positions representative studies by the decision they optimize, the endpoint they measure, and the energy boundary they adopt.

This study operates at the architecture-selection level. The comparison follows identical acquired records through finite archives, vertical ingress, gateway storage, vehicle carriage and surface receipt. A predefined parameter search separates tuning from independent evaluation, and an adaptive hybrid policy tests report-driven collection within a finite action catalogue. Complete-observation deadlines and explicit energy owners establish whether lower submerged draw preserves the required information service. Transfer contexts test the dependence on load, geometry, storage and contact quality. The resulting contribution is a reproducible deployment comparison and a diagnosis of its binding constraints; optimal control over arbitrary routes and schedules remains an open problem. Prior work establishes the feasibility of each component; the following sections compare the two complete service chains under one common operational workload.

## 3. Architecture and Decision Problem

### 3.1. Operational Boundary and Record Lifecycle

**Figure 1:**
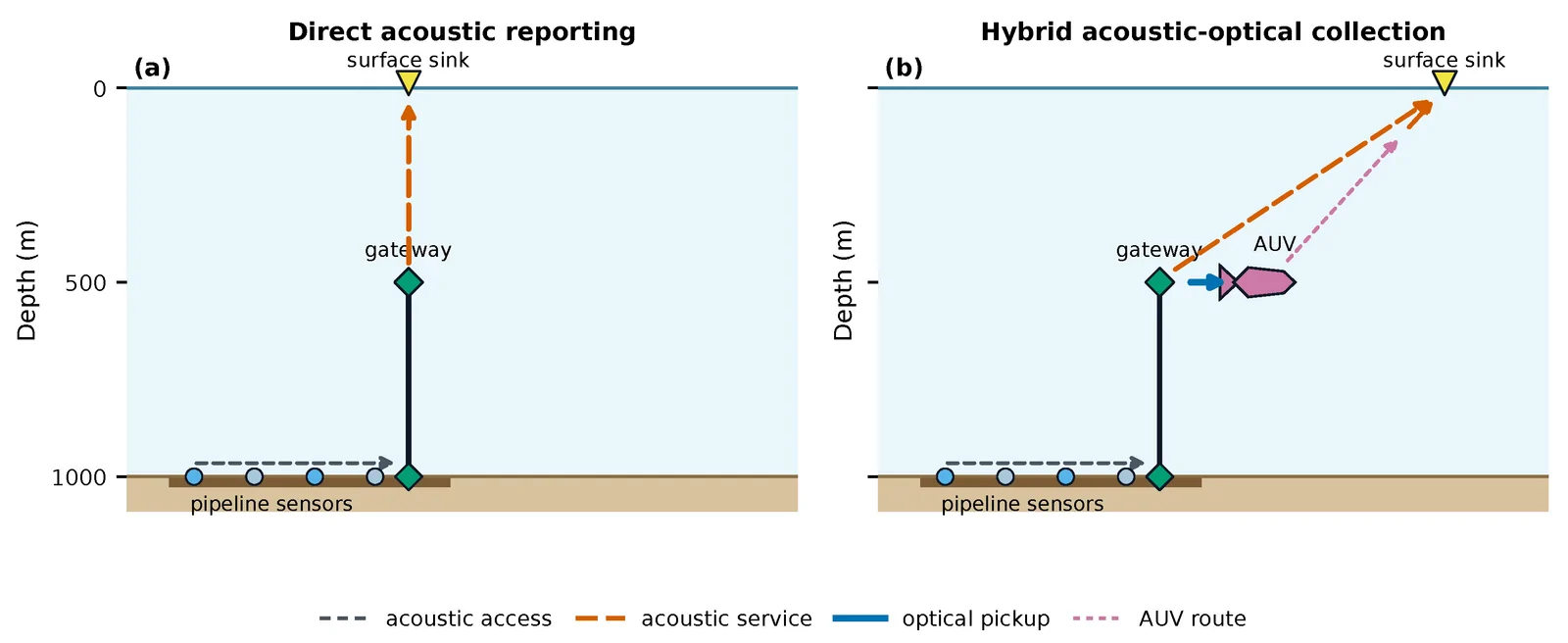
Operational alternatives evaluated in this study. (**a**) Direct priority sends every gateway record acoustically to a surface station. (**b**) Hybrid priority preserves acoustic access for alarms and aging status records, while an AUV retrieves routine records optically and later transfers them to the surface. The alternatives share the same traffic, priorities, gateways, surface stations, and geometry.

Figure 1 defines the two gateway-to-surface service chains. Seabed sensors and relays first bring alarm and status records to eight elevated collection gateways. High-volume inspection instruments are paired with those gateways through finite vertical ingress links. Acquisition, local archival storage and gateway admission are included in the reporting boundary. A record is delivered only after complete reassembly at a surface station. Gateway arrival and AUV pickup remain intermediate states. For raw measurements, timely recovery additionally requires every chunk of an acquisition window to arrive before its deadline.

Direct reporting sends every gateway record acoustically to a reachable surface station. Hybrid reporting preserves the acoustic path for urgent records but assigns routine records to scheduled optical pickup. A successful contact moves the record into the AUV carry queue, and the record remains incomplete until the vehicle finishes the modeled surface transfer. Both alternatives use the same pipeline, gateways, surface stations, offered traffic, class priorities, and available retry choices. The comparison therefore changes the service path, not the workload or supporting infrastructure. This matching isolates the service path as the sole source of any observed difference between the two chains.

### 3.2. Compared Service Configurations

Table 2 identifies the policies evaluated under the common resource model. Direct priority (D-P) provides the acoustic baseline. Static hybrid (H-S) adds a prescribed collection mission; adaptive hybrid (H-A) selects collection actions using received gateway reports. Each policy is evaluated over its declared parameter grid before held-out comparison. The FIFO control changes gateway eviction only, while retaining the same service-priority ordering.

The workload distinguishes alarms (HIGH), status updates (MEDIUM), and routine inspection records (LOW). Alarms have the strictest deadline and retain prompt acoustic access in both priority architectures. Status records receive an initial opportunity for AUV pickup in hybrid service and later fall back to the acoustic path. Routine records remain acoustic in direct service and use AUV pickup in hybrid service. This class mapping makes the intended operational exchange explicit: scheduled collection can protect alarm service and reduce gateway transmission, but it introduces waiting and an additional completion stage for less urgent data.

### 3.3. Decision Criteria

The architecture is judged on complete surface delivery, alarm and status timeliness, complete raw-window recovery, the location of retained records, and energy under a stated deployment boundary. Continuous source production is observed over a common horizon, while hybrid service receives one finite vehicle mission. A policy must satisfy mission feasibility and the service requirements before its energy can support an equal-service comparison. Independent confirmation and controlled changes to deployment conditions test the selected policies. With the alternatives and criteria defined, the following sections test them under a matched operational workload.

## 4. Materials and Methods

### 4.1. Reference Deployment and Workload

The reference corridor contains 180 nominal positions separated by 70 m over 12.6 km, consistent with role-based linear pipeline architectures [7,9]. Every fifth position acts as a relay, giving 144 traffic-generating sensors and 36 relays. Eight relay positions connect to elevated gateways at 500-meter depth, and 15 surface stations provide direct or terminal reception opportunities along the corridor. This dense fixed layout gives direct reporting frequent surface receivers and forms a demanding baseline for the added value of vehicle collection.

Alarm and status arrivals follow independent aggregate Poisson streams of 0.005 and 0.015 record/s, with 64- and 256-byte payloads distributed among the 144 sensing nodes. Each of the eight instruments acquires six motion channels at 10 Hz over successive five-minute windows. Six-axis acquisition and 10-Hz sampling are documented SMART options [34]; the 16-bit export format and deadlines are study choices. A window contains 6×10×300×2=36,000 payload bytes, transported in 25,000- and 11,000-byte chunks with a common window identity. Releases are staggered between sites and occur after acquisition finishes. The alarm, status and raw-window deadlines are 120, 600 and 30,000 s. Matched random streams preserve arrival times, classes, and sources across compared configurations. They also preserve acquisition times, raw-window identities and contents. Production continues to T=91,798.030 s, approximately 25.5 h, including after vehicle return.

### 4.2. Service and Communication Representation

Each instrument has a 1 GiB archive allocation. Both endpoints of its vertical link have 64 KiB staging; the gateway has a 16 MiB persistent outbox with 64 bytes of bookkeeping per stored chunk. The vehicle also has a finite 16 MiB allocation, with control and receipt reservations deducted. These are explicit study allocations. The documented 32 GB SMART archive provides a storage-scale reference, separate from sensing-node RAM [34]. Retained, unconfirmed copies consume their owner’s storage. A powered 9600-bit/s serial-service abstraction connects each instrument to its gateway, using ten wire bits per byte. A returned cable acknowledgement ends the source admission wait after durable gateway acceptance; its acquisition backup remains under the finite retention rule.

In hybrid service, HIGH records gain acoustic access at age 2 s, while MEDIUM records request fallback at age 60 or 120 s if they still await pickup. A shared one-credit token bucket regulates that fallback. Refill rates of 0.015, 0.020 and 0.030 record/s test equality with the mean status arrival rate and two levels of additional capacity. Acoustic retry limits of zero, one and three retries are varied for all policies; the budget applies over each acoustic leg’s lifetime.

The event-driven model represents propagation delay, spatial interference, distance-dependent reception, fragmentation, retries, reassembly, optical contact, vehicle storage, and final surface service. Acoustic service uses a 6 kbit/s rate, anchored to a documented Modem 6 telemetry mode (Sonardyne International Ltd., Yateley, UK) [35], with a 27 kHz loss proxy and 60 dB synthetic margin. Optical pickup uses a 30 m contact region and a 2.5 Mbit/s service scale, with BlueComm 200 UV electrical-power anchors (Sonardyne International Ltd., Yateley, UK) [36]. Nominal optical frame success is one within eligible contacts; admission, contact duration, power and acknowledgement failures remain possible. Actual acoustic control opens the listening window and confirms reception; startup, waiting and unsuccessful activity consume energy. The exact link, framing, retry, contact, and power functions are documented in Appendix A; the main analysis uses the same functions and service limits for every alternative.

The vehicle uses the HUGIN 6000 67 kWh configuration (Kongsberg Discovery, Kongsberg, Norway) as its battery reference, with published endurance of 65–76 h at 3 kn with sensors operating [37]. The study fixes 3 kn in calm water, a 900 W operating baseline, a 20% reserve and a 600 s externally charged recovery allowance. Additional communication draws from the same finite battery. Routes retain continuous position and heading, finite-radius turns, bounded depth and pitch, and return to the original submerged rendezvous. A 100-meter-radius service circle takes approximately 407.1 s at that speed; discretized service allowances enter both time and energy budgets.

H-S follows a prescribed visit order. H-A evaluates one or two pickups followed by offload and return using time-stamped acoustic reports actually received. Reports are attempted every 300 s and remain usable for at most 1200 s; an unreported remote state is unknown. Actions account for complete windows, carry storage, offload service, reserve and coverage. They first minimize projected late windows, then favor timely payload per unit mission time, with deterministic tie-breaks. All currently carried data must fit the first subsequent offload, an explicit conservative policy restriction. A feasible return suffix is retained if no new action is admitted. Mission feasibility requires visiting all eight sites within the active-mission coverage bounds. This finite catalogue defines the tested adaptive controller.

### 4.3. Deadline and Energy Measures

Deadline performance includes only observations old enough to receive their complete deadline window. For class *C* with deadline $d_{C}$ and run end *T*, let $N_{C}^{\mathrm{obs}}$ count offered observations whose deadline origin is no later than $T-d_{C}$, and let $D_{C}^{\mathrm{on}}$ count members delivered to a surface station by their individual deadline. The origin is source creation for alarms/status and the oldest sample for a raw window; LOW delivery requires all its chunks. Losses before gateway admission remain in the offered denominator. The miss ratio is

(1) $${\hat{m}}_{C}=1-\frac{D_{C}^{\mathrm{on}}}{N_{C}^{\mathrm{obs}}},\;N_{C}^{\mathrm{obs}}>0.$$

This denominator prevents recently generated records from being labeled late before their deadline has elapsed. Mean delay is calculated for delivered records and is always interpreted with the corresponding delivery count. The reference service targets are at most 1% misses for HIGH traffic and 5% for MEDIUM and complete-window LOW traffic. Later windows are right-censored for the miss ratio; they remain in delivery and terminal-state accounts and may already have arrived.

Energy is reported through two deployment accounts. Let $E_{\mathrm{fixed}}$ contain consumption of the submerged sensors, relays, gateways and acquisition/logging instruments; $E_{\mathrm{surface}}$ be the surface-station consumption; and $E_{\mathrm{vehicle}}$ be the vehicle’s operating, communication and recovery energy. The complete system account is

(2) $$E_{\mathrm{total}}=E_{\mathrm{fixed}}+E_{\mathrm{surface}}+E_{\mathrm{vehicle}}.$$

Direct reporting has zero AUV terms. Fixed submerged-equipment and full-system energy are reported separately so that a local communication saving cannot be mistaken for a complete mission-energy saving. Elapsed interface states and finite battery owners charge failed transmissions, corrupted or overheard reception, listening, startup and cable activity independently of useful delivery.

### 4.4. Matched Experiments and Observation Horizons

Table 3 separates parameter selection from independent confirmation and controlled deployment changes. The declared grid contains 3 direct configurations and 18 for each hybrid policy: three retry limits for all policies, combined with two fallback ages and three refill rates for hybrid service. Every candidate uses the same five tuning seeds. A representative must first pass the mission, trajectory, storage and conservation checks on every run, and meet the mean service thresholds. Among qualifying candidates, selection minimizes mean fixed submerged-equipment energy, then mean total energy, with a fixed identifier tie-break. If service is infeasible, the diagnostic minimizes the largest normalized service violation among mission-admissible candidates. A family without a mission-admissible candidate has no held-out representative. Within every comparison, matched random streams preserve arrival times, class labels, and sources; each service then develops its own channel and queue history.

The selected D-P representative and H-S diagnostic remain frozen for the remaining tests. No H-A candidate met mission feasibility; its 90 tuning results are retained and its dependent tests are not reassigned to further search. All performance runs retain the common T=91,798.030 s source-production horizon. Hybrid mission completion does not stop demand. A separate set of 19 network verification runs, including preserved failed and interrupted checks, is excluded from performance estimates.

Table 4 defines six single-factor transfer contexts. The longer corridor keeps the same vehicle and observation duration; the optical perturbation tests link reliability within contacts. The summary-only control instead supplies one 256-byte record per site per hour, with a 3600 s window and a deadline starting at that hour’s beginning. It changes the information product and temporal detail, rather than compressing the same five-minute acquisition at equal freshness. The FIFO control changes only the gateway eviction order under the constrained outbox. The immediate-fallback control changes only the H-S MEDIUM waiting gate.

### 4.5. Outcomes and Statistical Comparisons

Complete-window recovery counts acquisition windows received in full at the surface. Deadline ratios use the mature groups defined in Equation (1). Packet-lifecycle traces connect generation with complete surface reception and separately with sender confirmation, and the terminal ledger separates seabed-access loss, deadline expiration, admission loss, ongoing service, gateway storage, and AUV storage. This distinction prevents an acknowledgement or an intermediate pickup from being counted as a second delivered observation. The energy outcomes use the two boundaries in Equation (2). Together, these measures connect application outcomes to the service mechanism that produces them.

For the reference comparison, the primary effect is the paired within-seed hybrid-minus-direct-priority difference. Independent runs are the replication units. Rates are means of run-level ratios; paired energy reductions average the per-run relative differences. Exploratory 95% percentile intervals use 10,000 bootstrap samples of paired run differences with a fixed analysis seed, following independent-replication principles [38]. Five-run transfer intervals have limited precision, and multiple comparisons have no simultaneous coverage guarantee. The FIFO control shares seeds with the 1 MB baseline. Immediate fallback uses seeds 321–325 rather than confirmation seeds 311–320; its nominal comparison is descriptive, not a paired causal effect. Tuning means are reported separately from confirmation estimates.

### 4.6. Event Accounting and Reproducibility

Each experiment follows every record through acquisition, finite ingress, gateway arrival, acoustic service, optical pickup, AUV carriage, and complete surface delivery while representing vehicle motion, spatial interference, deadlines, retries, and energy. The implementation uses ns-3.40 with AquaSim-NG v1.1 [39,40]. Conservation checks assign every generated record to delivery, expiration, access loss, ongoing service, or storage. Additional checks cover finite memory, complete-window accounting, actual reverse acknowledgements, state-energy ownership, continuous routes and mission return. A terminal transfer failure is a loss; an unresolved retained copy is backlog. Duplicate sender copies are excluded from unique-record totals while remaining in physical storage and energy accounts. The supplementary package contains the simulator, versioned execution environment, experiment commands, run-level outputs, logs, and analysis code. The development repository is available at https://github.com/Aissata98/ns3-new, accessed on 20 September 2026.Archived inputs, arguments, source identities, execution receipts and selection decisions identify the reported campaign. A rebuild on another platform produces a separately recorded executable identity.

## 5. Results

### 5.1. Complete-Window Recovery Distinguishes the Deployments

The predefined search evaluated 39 configurations over five common seeds, giving 195 runs. Among three direct configurations, D-P with three retries alone met the mean service targets. All 18 static-hybrid configurations passed the mission checks, but none met the raw-window target. The retained H-S comparator is therefore the declared best service-violation diagnostic: zero retries, a 120 s status age gate and a 0.030-record/s fallback refill. None of the 18 adaptive configurations was mission-feasible across its five seeds; H-A consequently has no held-out representative. These failures remain part of the comparison.

Independent confirmation comprised 20 runs: ten new common seeds for each retained policy. Direct reporting met the mean alarm, status and raw-window targets, recovering all 1648 deadline-eligible raw windows per run on time. Static hybrid recovered a mean of 13.8, or **0.84%**; its mean alarm misses also exceeded the 1% target, while status misses remained below 5% (Table 5). This contrast concerns complete observations delivered to the surface, not only eventual receipt of individual packets.

### 5.2. Fallback Timing and Deployment Conditions Define the Trade-Off

Fallback refill was varied at and above the offered mean status rate. At zero retries, raising the refill from 0.015 to 0.020–0.030 record/s substantially reduced status misses; complete raw-window recovery remained below 1% across all six age/refill combinations (Table 6). The highest tested refill was selected for the static-hybrid diagnostic, but this forwarding change neither adds a vehicle mission nor increases its final transfer allowance.

The frozen policies were then tested in six changed deployment contexts, with five common seeds per context and policy (60 runs; Table 7). Direct reporting met the mean service targets under higher event load, the longer corridor, the smaller outbox and the optical disturbance. Its advantage depended on infrastructure: with five surface receivers, timely raw-window recovery fell to **37.50%**, and both policies missed about 68% of alarms. Doubling raw sample width preserved direct raw-window recovery but increased its alarm and status misses to **2.62%** and **5.99%**. Hybrid had lower alarm/status misses in that context, yet recovered only 0.52% of raw windows on time. Neither policy met the combined requirement there.

**Figure 2:**
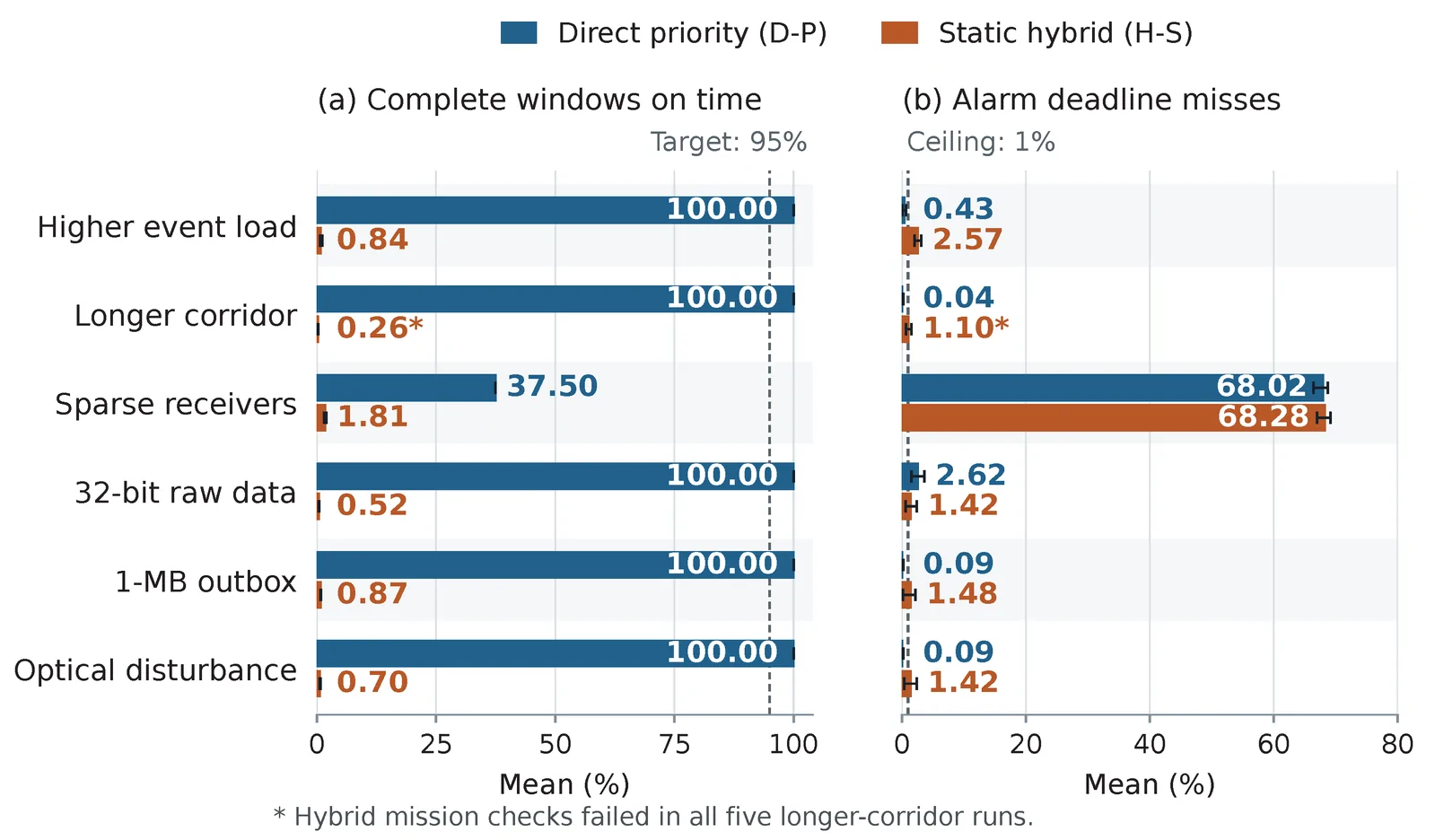
Transfer across six contexts, five common seeds per policy and context. Horizontal bars and labels show mean percentages; whiskers show the observed minimum–maximum, not confidence intervals. Dashed lines mark the 95% timely-recovery target and 1% alarm-miss ceiling. Asterisks identify failed static-hybrid mission checks in all five longer-corridor runs.

No transfer context made the static-hybrid diagnostic service-feasible. In the longer corridor, its mission checks failed, and only 0.26% of eligible raw windows arrived on time. Figure 2 shows the measured variation and the prescribed targets, retaining the infeasible mission outcomes.

### 5.3. Continuous Production Exposes Collection Constraints

Static hybrid visited all eight sites and completed its finite mission after approximately 30,450 s, while sensing continued throughout the 91,798 s observation. Before mission completion, 805 mature windows had already finished acquisition. Nevertheless, a mean of only 13.9 complete windows reached the vehicle and 13.8 reached the surface (Table 8). The main complete-window deficit was already present before final offload, despite zero nominal gateway-capacity evictions. Repeated optical receptions and expired acknowledgement waits further show why received chunks cannot be equated with useful complete-window collection. A timeout identifies an expired wait, not a proven physical loss of an acknowledgement.

Accepted wake sessions are not geometric contacts or measured usable contact duration. The traces omit unattempted range/window gate exits, so the reported stages do not constitute an exhaustive failed-contact decomposition.

The adaptive search failures have two observed admission mechanisms. In 39 of its 90 runs, further service was rejected because the projected onboard load required 419.7–760.6 s of offload, exceeding the ordinary 407.1 s allowance; the conservative planner requires that load to fit the first offload. The other 51 runs stopped admitting pickups after five sites when the next first visit could no longer meet the 30,000 s coverage bound. All returned with 88.62–98.82% of battery capacity remaining. These outcomes identify restrictions of the tested planning rule and route history, rather than battery depletion or an optimality claim about hybrid collection.

### 5.4. Energy Savings Accompany Unequal Information Service

**Figure 3:**
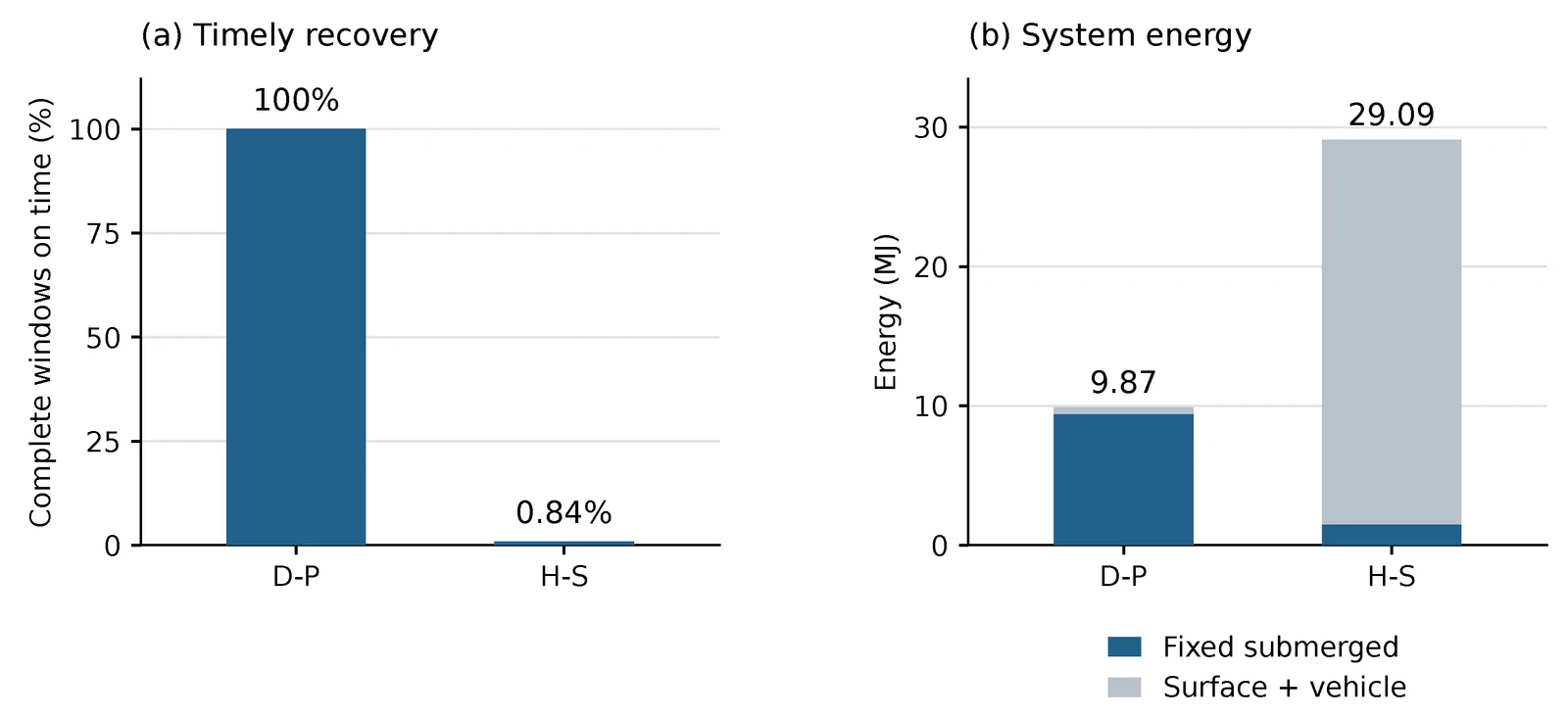
Confirmation service and energy under the common observation horizon. (**a**) Complete eligible raw windows received on time. (**b**) Fixed submerged energy and the additional surface/vehicle account. One finite hybrid mission accompanies continuing production; the compared information service is unequal.

Hybrid reporting reduced fixed submerged-equipment energy by **83.93%** in confirmation, with an exploratory paired 95% bootstrap interval of 83.90–83.97%. Including surface equipment and the vehicle increased the total energy by **194.56%** (194.49–194.62%). This reduction in fixed-equipment draw accompanies a **99.16-percentage-point** loss in timely complete-window recovery, so it is not an efficiency gain at equivalent service (Figure 3). The vehicle consumed approximately 7.62 kWh, or 11.37% of its initial battery; the prescribed mission ended with substantial reserve while production continued.

The remaining controls comprised ten summary-product runs, ten eviction-ablation runs and five immediate-fallback runs (Table 9). Hourly summaries change the information product; direct reporting recovered every eligible summary and hybrid recovered 11.59%, still below the 95% target. FIFO changes only gateway eviction, leaving priority service unchanged. Relative to the paired 1 MB priority-eviction runs, direct metrics were unchanged and hybrid timely-window recovery increased by only 0.012 percentage points (exploratory paired 95% interval 0–0.036). Immediate hybrid fallback produced 4.48% status misses and 0.81% raw-window recovery. Its seeds differ from nominal confirmation, so this last comparison is descriptive, without a paired nominal gate-effect estimate. None of these controls made the tested finite static mission meet the combined service requirement.

## 6. Discussion

Together, the results define a conditional operating boundary. Hybrid pickup lowers fixed-submerged equipment draw, but the selected static hybrid service recovers very few complete raw observations on time. The comparison depends on the acquired information product, deployment geometry and finite vehicle service, as well as the accounting boundary.

### 6.1. What the Observation Horizon Reveals

The observation follows continuously operating instruments served by one bounded vehicle mission. Generation continues for 91,798 s, while the reference hybrid mission ends after approximately 30,450 s. Direct priority delivers 100% of deadline-eligible raw windows on time; the static hybrid diagnostic delivers 0.84%. A window counts only when all its chunks reach the surface, and eligibility requires its full deadline to fall inside the observation. This comparison therefore measures timely recovery under the declared mission allocation. Finite generation followed by a drain interval answers the complementary question of eventual recovery once new demand stops. The submitted finite-drain and repeated-patrol analyses are preserved in Supplementary Materials File S1 under their original workload and service assumptions; their record-level estimates are kept separate from the complete-window evaluation. Repeated sorties would add collection opportunities, energy and recovery requirements and require a separately specified operating schedule.

Mission duration is part of this explanation, but it does not explain the entire deficit: 805 deadline-eligible raw windows have already been acquired before the reference mission ends. The vehicle consumes 11.37% of its initial battery, so this endpoint is a prescribed mission boundary rather than battery exhaustion. Complete-window service also distinguishes useful recovery from isolated chunk reception. The resulting deployment criterion is the number of complete observations delivered on time, evaluated together with the resources committed to delivering them.

### 6.2. Mechanisms Behind the Trade-Off

The reference static mission completes nine visits across eight collection sites. Across ten confirmation runs, the AUV receives a mean of 34.7 unique raw chunks, forming 13.9 complete windows onboard; 13.8 complete windows reach the surface. The main loss of complete-observation service therefore precedes successful collection in this reference instance. Acoustic wake-up, optical reception and custody confirmation are separate events: a received chunk can remain unconfirmed at its gateway and trigger a duplicate transmission. The tested fallback rates span 0.015, 0.020 and 0.030 records/s. Increasing that reserve addresses status forwarding within the tested policy, while raw collection still depends on finite visits and successful transfers.

The adaptive family supplies a further policy-level diagnosis. None of its 18 tested configurations provides an admissible representative across the five tuning seeds. In 39 missions, a conservative first-offload admission rule rejects further service; in the other 51, the route consumes the slack needed to cover all collection sites. Substantial battery energy remains in both cases. These outcomes identify offload allocation and coverage reservation as specific controller improvements, rather than an upper bound on hybrid performance. Exhausting the declared grid establishes a best tested representative under its selection rule, while global optimization and a numerical comparison with published learning controllers require a wider, matched implementation. This diagnosis complements optimization and learning studies that select routes, links, and schedules within AUV-assisted systems [13,14,15,16,31]: the matched baseline identifies where an adaptive policy must create additional value and which outcomes must remain visible.

### 6.3. Deployment Decision Boundary

These mechanisms and their energy implications define the deployment recommendation. **Direct priority** is the service-feasible reference choice for the specified acquisition product and deployment. Its advantage is conditional: the sparse-receiver context degrades both services, and the larger raw-sample encoding context preserves direct raw-window completion while increasing alarm and status deadline misses. **Hybrid priority** reduces local equipment draw in the reference case, but this benefit accompanies substantially less delivered information. A useful hybrid deployment therefore requires a collection schedule and information product that meet the service target. Reusing an independently funded inspection patrol would additionally require a causal account of added detours, waiting and payload service.

The same boundary resolves the apparently different energy results. Energy at fixed submerged equipment falls from 9.433 to 1.516 MJ, with a mean paired reduction of 83.93%. Including surface equipment and the complete deployed vehicle mission changes the totals to 9.875 and 29.087 MJ, respectively, a mean paired increase of 194.56%. These values describe the same observation horizon and different amounts of completed service; the local reduction is therefore not an equal-service efficiency gain. The vehicle baseline, communication and recovery allowance are included in the total, while an incremental patrol-reuse account requires a separately specified deployment.

The distinction between incremental reporting cost and full mission cost remains useful for an existing inspection patrol. If its route, duration and operating load remain unchanged by collection, the reporting account is $E_{\mathrm{inc}}=E_{\mathrm{fixed}}+E_{\mathrm{surface}}+E_{\mathrm{comm},\mathrm{AUV}}$, where $E_{\mathrm{comm},\mathrm{AUV}}$ is onboard communication energy and independently required vehicle operation is allocated to inspection. Any collection-induced detour, waiting or recovery cost must also enter this incremental account. A matched patrol with and without collection is needed to estimate that added cost. The present runs support the full deployed-mission account; the submitted incremental estimate is preserved only within its original assumptions in Supplementary Materials File S1.

### 6.4. Scope, Validity, and Field Transfer

The evidence applies to a 12.6 km reference pipeline with synthetic alarms and status reports, sampled raw measurements, finite byte-limited storage and the tested communication and mission settings. Longer and sparse-receiver contexts, increased event load and raw-sample width, reduced gateway storage and an optical-success perturbation test specified departures from that reference. Documented acquisition, modem rates, optical electrical draw and vehicle battery size anchor the resource scales. Range gates, channel loss mapping, memory allocation and calm-water vehicle power remain explicit engineering assumptions. Component tests and conservation checks establish internal execution and accounting consistency; integrated hardware measurements are needed to calibrate those assumptions. Persistent archive capacity alone does not establish available processor memory or lower-layer queue capacity.

Field transfer requires measured bathymetry, sound-speed profiles, multipath, interference, modem error curves, optical water properties, vehicle power, and the selected surface interface. Recovery, recharge, support-vessel time, and receiver installation also enter a project-level energy and cost account. These measurements calibrate the decision boundary without changing the central test: compare complete direct and hybrid service chains under the same workload and attribute vehicle cost according to the mission that causes it.

Currents alter travel time and return reserve; position and attitude errors affect optical alignment, while clock and localization errors affect rendezvous [33]. The optical-success perturbation changes reception inside the modeled contact region. A field-calibrated extension must model these hydrodynamic and navigation processes explicitly before interpreting the results as installation-level performance predictions.

## 7. Conclusions

This matched end-to-end study compares tuned direct and AUV-assisted hybrid reporting for subsea pipeline monitoring under finite resources. Complete measurement windows are followed from acquisition to surface receipt, with deadlines, storage, acknowledged transfers and vehicle operation included. Independent confirmation and transfer contexts distinguish reference service feasibility from behavior under changed deployment conditions. Separate reporting of fixed-submerged and total system energy connects each energy result to both the deployment and the information service that produces it.

For the reference deployment, direct priority delivers **100%** of deadline-eligible raw windows on time, compared with **0.84%** for the selected static hybrid diagnostic. Hybrid fixed-submerged draw is **83.93%** lower, while its total system draw is **194.56%** higher; the local saving accompanies incomplete service. One finite mission serves continuously operating instruments, and that mission ends with substantial battery reserve. The adaptive catalogue fails its coverage requirements, identifying conservative offload admission and coverage slack as controller limitations. These results favor direct priority for the tested reference requirements and identify concrete conditions a revised hybrid service must satisfy. The practical contribution is an end-to-end acceptance test: evaluate complete timely observations, feasible collection and total deployed resources together, rather than infer deployment value from a local communication saving.

## Figures and Tables

**Table 1:** Representative underwater collection and pipeline-monitoring studies, with recent resource-allocation and localization context, ordered by publication year and positioned by decision, evaluation endpoint, and energy boundary.

Study	Year	Architecture/Decision	Evaluation Criterion	Energy Boundary
Janardanan Kartha and Jacob [17]	2015	Direct/multihop versus mobile-sink acoustic collection	PDR; queue; delay to mobile sink	Sensor energy/lifetime; mobile-sink endpoint
Zheng et al. [8]	2017	Pipeline AUV tour and resurfacing	Mean reporting delay to surface	Route and resurfacing focus
Wang et al. [24]	2017	Hybrid optical–acoustic versus acoustic network	Connectivity; loss; end-to-end delay	Node energy balance and lifetime
Fang et al. [12]	2021	Multi-AUV limited-service collection	Peak AoI; stationary queue	Patrol and vertical-motion energy
Liu et al. [30]	2022	Within-architecture urgent forwarding and AUV retrieval	Value, timeliness, and energy efficiency	Node communication and AUV route
Ahmed et al. [9]	2022	AUV routing on a linear pipeline	PDR; end-to-end delay; throughput	Traffic-load evaluation
Bu et al. [13]	2023	Within-architecture trajectory and acoustic/optical selection	Importance- and size-weighted AoI	Node transmission and AUV-motion energy
Zhu et al. [28]	2024	Priority- and void-aware hybrid routing	Priority; PDR; delay	Forwarding energy; network lifetime
Kasparavičiūtė et al. [23]	2025	Resident-AUV route and reprioritization	Route time; priority completion	Battery threshold; docking and recharge
Wei et al. [16]	2025	Channel-aware AUV path planning	PDR; collection rate under loss	Node communication and AUV-motion energy
Weng et al. [29]	2025	Optical AUV tracking and seafloor-platform pickup	Link alignment; sea-trial retrieval	Tracking-energy objective
Lee et al. [31]	2026	Within-architecture collection/relay scheduling to a buoy	Buffer-coupled peak AoI; delivered packets	End-to-end relay and AoI focus
Zhang et al. [11]	2026	AUV–USV inspection with terminal offload	Final buffer; upload success; mission time	Motion-energy proxy; terminal phase included
Kang et al. [33]	2026	Underwater clock calibration and localization	Clock and sensor-position uncertainty	Calibration/localization focus
He et al. [32]	2026	Aerial resource and trajectory control	Secure communication	Resource-allocation focus
**This study**	–	Direct-versus-hybrid architecture selection with finite vehicle service	Complete-window deadlines; backlog; feasibility	Fixed submerged; complete deployed system

**Table 2:** Service configurations and their role in the controlled comparison.

Policy	Routine-Record Path	Analytical Role
D-P	Acoustic surface service	Prioritized direct baseline.
H-S	Optical pickup and AUV carriage	Prescribed visit order with checked service and return legs.
H-A	Optical pickup and AUV carriage	Causal one- or two-site collection decisions.

**Table 3:** Executed evaluation matrix. Counts are total performance runs. Selection uses seeds 301–305, confirmation uses new seeds 311–320 and the remaining phases use seeds 321–325. The absent H-A representative leaves 55 dependent held-out cases explicitly unexecuted.

Experiment	Compared Configurations or Change	Runs
Parameter selection	All 39 configurations; five common seeds	195
Confirmation	Frozen D-P and H-S; ten new seeds each	20
Deployment transfer	Six contexts; D-P/H-S; five seeds each	60
Summary product	Hourly summary input; both policies	10
FIFO eviction	Same 1 MB outbox; both policies	10
Immediate fallback	H-S MEDIUM age gate set to zero	5
Total	Performance evaluation	**300**

**Table 4:** Single-factor transfer contexts. Unlisted nominal parameters and the source-production horizon remain fixed. MB denotes $10^{6}$ bytes.

Context	Change from the Reference Deployment
Event load	Double alarm and status arrival rates; raw acquisition unchanged.
Longer corridor	25.2 km, 360 positions, 15 collection sites, 29 surface receivers; same spatial densities.
Sparse surface	Five surface receivers instead of fifteen; unchanged ranges.
Raw encoding	32-bit rather than 16-bit samples: 72,000 bytes per window.
Gateway storage	1,000,000-byte rather than 16 MiB outbox.
Optical disturbance	DATA-frame success probability 0.9 rather than 1 within eligible contacts.

**Table 5:** Independent confirmation over ten common seeds. Entries are means of run-level metrics. HIGH/MEDIUM are deadline-miss percentages; LOW is the percentage of complete eligible windows received on time. Fixed energy covers submerged equipment; total energy also includes surface equipment and the vehicle.

Policy	HIGH Miss (%)	MEDIUM Miss (%)	LOW on Time (%)	$E_{\mathrm{fixed}}$ (MJ)	$E_{\mathrm{total}}$ (MJ)
D-P	**0.28**	**0.93**	**100.00**	9.433	**9.875**
H-S	1.42	4.80	**0.84**	**1.516**	29.087

**Table 6:** Static-hybrid training sensitivity at zero retries, five common seeds per row. HIGH/MEDIUM are deadline-miss percentages; LOW is timely complete-window recovery. All 39 configurations remain in the artifact.

Age (s)	Refill (Record/s)	HIGH Miss (%)	MEDIUM Miss (%)	LOW on Time (%)
60	0.015	0.95	27.11	0.90
60	0.020	1.04	4.80	0.91
60	0.030	0.99	4.63	0.87
120	0.015	1.00	31.60	0.92
120	0.020	0.86	4.91	0.97
120	0.030	0.95	4.76	0.98

**Table 7:** Frozen-policy transfer, five common seeds per context. Entries are run means. HIGH/MEDIUM are deadline-miss percentages; LOW is timely complete-window recovery. The asterisk identifies failed hybrid mission checks in all five longer-corridor runs; accounting audits passed.

Context	Policy	HIGH Miss (%)	MEDIUM Miss (%)	LOW on Time (%)	$E_{\mathrm{total}}$ (MJ)
Higher event load	D-P	0.43	2.28	100.00	10.000
	H-S	2.57	12.44	0.84	29.178
Longer corridor	D-P	0.04	0.75	100.00	19.365
	H-S *	1.10	4.42	0.26	29.347
Sparse receivers	D-P	68.02	69.32	37.50	4.131
	H-S	68.28	70.11	1.81	29.850
32-bit raw data	D-P	2.62	5.99	100.00	18.109
	H-S	1.42	4.29	0.52	29.148
1 MB outbox	D-P	0.09	1.02	100.00	9.874
	H-S	1.48	4.42	0.87	29.253
Optical disturbance	D-P	0.09	1.02	100.00	9.874
	H-S	1.42	4.47	0.70	29.077

**Table 8:** Static-hybrid collection stages over ten confirmation seeds. Ranges are per-run minimum–maximum. Sessions contain newly received optical data and are not a count of geometric contacts.

Observed Stage	Mean	Range
Completed visits/unique sites	9/8	9/8
Sessions receiving new optical data	9.8	9–11
Unique LOW chunks received optically	34.7	27–42
Unique LOW payload received optically (kB)	672.9	535–798
Complete raw windows onboard	**13.9**	10–18
Complete raw windows received at surface	**13.8**	9–18
Duplicate optical receptions, all classes	17.5	15–18
Optical acknowledgement wait expirations	25.6	24–26

**Table 9:** Controlled experiments, five runs per row. HIGH/MEDIUM are deadline-miss percentages. LOW denotes eligible hourly summaries in the first two rows and raw windows otherwise. FIFO changes eviction only; immediate fallback has no paired nominal counterpart.

Control	Policy	HIGH Miss (%)	MEDIUM Miss (%)	LOW on Time (%)	$E_{\mathrm{total}}$ (MJ)
Hourly summary	D-P	0.00	0.19	**100.00**	1.603
	H-S	1.21	4.28	**11.59**	28.992
FIFO, 1 MB	D-P	0.09	1.02	**100.00**	9.874
	H-S	1.44	4.41	**0.89**	29.253
Immediate fallback	H-S	1.37	4.48	**0.81**	29.088

## Data Availability

A supplementary reproducibility package containing the simulator source, versioned build environment, experiment workflows, analysis code, execution logs, per-run time series, and data underlying the reported results accompanies the manuscript. The development repository is available at https://github.com/Aissata98/ns3-new, accessed on 27 September 2026. The accompanying revision archive identifies the exact model and results reported here; historical experiments are retained separately.

## Supplementary Materials

The following supporting information can be downloaded at: https://www.mdpi.com/article/10.3390/s26196337/s1, Supplementary File S1 preserves the submitted-model result tables, their original observation protocols and a mapping to the revised analyses. These historical estimates are distinguished from the revised finite-resource evaluation; Table S1: Finite-traffic reference results for 25 kB routine records at λ = 0.05 records/s, followed by queue drain and averaged over 20 common seeds. The primary effect is hybrid priority minus direct priority; percentage effects are percentage points; Table S2: Sensitivity to the synthetic acoustic margin. D/H denotes direct priority/hybrid priority; Δ is hybrid minus direct. Values use 10 matched seeds at each margin; Table S3: Hybrid sensitivity to the MEDIUM fallback age gate. Intervals are Student 95% intervals over 20 independent runs; pooled ratios provide an event-level audit of the run-level inference; Table S4: Continuous-arrival service over five complete post-warm-up patrol cycles. Metric entries are run means with 95% Student half-widths over five runs. A/D/S/P are pooled terminal seabed-access losses, deadline expirations, stored records, and pending transactions; every record passed admission; Table S5: Mean stored gateway-plus-AUV records at exact patrol-cycle ends. Trends are run means with 95% Student half-widths over five runs and use the five displayed cycle-end points; Table S6: Sensitivity of submerged-device energy to idle or hibernation power per nominal submerged position. Values compare hybrid priority with direct priority; 0.5 mW is the low-power reference input.

## Abbreviations

The following abbreviations are used in this manuscript:

AoI	Age of Information
AUV	Autonomous Underwater Vehicle
BSN	Basic Sensor Node
CI	Confidence Interval
DDN	Data Dissemination Node (collection gateway)
D-P	Direct reporting with priority
FIFO	First-In, First-Out
H-A	Hybrid reporting with the adaptive catalogue
H-S	Hybrid reporting with the static mission
HIGH	Alarm traffic with the strictest deadline
LOW	Routine bulk records eligible for delayed collection
MAC	Medium Access Control
MEDIUM	Status traffic with an intermediate deadline
PDR	Packet Delivery Ratio, as reported by prior studies
RDR	Record Delivery Ratio

## Appendix A. Implemented Service, Link, and Energy Functions

This appendix documents the exact simplified functions used by the event-driven evaluation, keeping the main system narrative focused on the deployment study. The equations make distance, payload, contact, and energy assumptions auditable and define the study scenario. ALOHA contention, serialization, timeouts, retransmissions, and reassembly remain discrete events. The AquaSim RangePropagation module filters each frame by the transmitting PHY range before MAC collision processing, creating spatial reception neighborhoods. The acoustic probability below then supplies an independent distance-dependent reception draw after successful contention of an addressed frame.

Table A1 identifies the documented equipment anchors. Table A2 separately lists the engineering settings, including finite storage, ingress and mission rules.

**Table A1:** Documented equipment anchors used to define the study instance. Allocations and protocol parameters are specified separately.

Reference	Documented Properties Used
SMART Type 8316	Six motion axes; 10 Hz option; 32 GB archive; typical 200 mW logging; 100 Ah, 15 V battery; acoustic reporting and supported optical harvesting [34].
Modem 6	TS4, TS5 and TS7 user-data rates of 3, 6 and 9 kbit/s; the 6 kbit/s service scale is selected [35].
BlueComm 200 UV	Type 8361; 2.5–10 Mbit/s; 405 nm emitter; 30 W emitter and 10 W receiver electrical draw; 24–36-V supply [36].
HUGIN 6000	67 kWh configuration; published endurance of 65–76 h at 3 kn with sensors operating [37].

**Table A2:** Principal engineering settings fixed before the revised policy comparison. MiB and GiB are binary byte units. Unless identified in Table A1, powers and allocations are study assumptions.

Component	Executed Setting
Acoustic service	27 kHz; 6 kbit/s; 60 dB margin; 512-byte fragments; ALOHA; fixed 300 m sensing/relay and 1000 m gateway/vehicle/surface gates.
Acoustic draw	Transmit 10 W; receive 2 W; awake idle 0.05 W. The operating policy is awake; the declared sleep state is 0.005 W.
Station batteries	Sensing node 100 Wh; relay, gateway and surface station 500 Wh each; instrument 1200 Wh, an assumed 80% usable fraction of its 1500 Wh nameplate reference.
Electronics	Sensing baseline 0.01 W; relay/gateway/surface baseline 0.05 W each; separate instrument logging 0.2 W.
Vertical ingress	9600 bit/s; 500-m dedicated service; propagation 2.5 μs; 10 ms admission; 1 ms turnaround; cable transmit/receive 0.2/0.1 W at each endpoint.
Finite storage	Archive 1 GiB; source/gateway cable staging 64 KiB each; gateway outbox and total vehicle allocation 16 MiB each; 64-byte record bookkeeping.
Optical session	30 m gate; 2.5 Mbit/s; 1 s startup; 30 s listening; 5 s polling; supply efficiency 0.9; frame success 1; three attempts; 8 MiB credit; 4096 receipt entries.
Control/timing	Control body at most 512 bytes; 30 s lifetime; 16 source and 16 receive slots; acoustic grace 12 s after local transmission completion; optical confirmation wait 12 s.
Vehicle	One finite mission at 3 kn; 900-W baseline; 67 kWh; 20% reserve; external recovery 600 s and 540 kJ.
Coverage/reports	Active-mission visit bound 30,000 s; reports every 300 s, usable for 1200 s; source production throughout 91,798.030 s.

For an acoustic physical fragment with payload *b* bytes and geometric distance *d* in meters, let $d_{a}=\max\left(d,1\right)$ and let *f* be in kHz. The implemented absorption, practical-spreading loss, simplified signal-to-noise quantity, and independent-fragment success probability are

(A1) $$\begin{array}{cc} \alpha (f) & =\frac{0.11f^{2}}{1+f^{2}}+\frac{44f^{2}}{4100+f^{2}}+2.75\times 10^{-4}f^{2}+0.003,\\ L_{a}\left(d,f\right) & =15\log_{10}\left(d_{a}\right)+\alpha \left(f\right)\frac{d_{a}}{1000},\\ \gamma_{a}\left(d,f\right) & =10^{\left(M-L_{a}\left(d,f\right)\right)/10},\\ p_{a}\left(d,b\right) & ={\left(1-\frac{1}{2}e^{-\gamma_{a}\left(d,f\right)}\right)}^{8b}. \end{array}$$

Here, α is in dB/km, $L_{a}$ and the reference margin *M* are in dB, and $p_{a}$ is applied independently to each physical fragment. The first line is the Thorp absorption law, the $15\log_{10}d_{a}$ term implements practical spreading, and the final line is the simulator-specific exponential bit-error model inspired by the form used for noncoherent binary FSK [4,41,42]. The quantity $\gamma_{a}$ is a simulator-specific signal-to-noise proxy. Payload bytes define the success exponent, while the 51- or 75-byte data header contributes to serialization time and energy. The transmit-power settings in Table A2 feed the energy ledger independently of $\gamma_{a}$ and $p_{a}$.

The revised comparison fixes f=27 kHz and M=60 dB. The documented Modem 6 rate anchors the service scale; the framing, contention and loss functions define the simulated realization.

Fragments carry at most 512 payload bytes. Data headers have 51 bytes for alarms/status and 75 bytes for raw data, including the latter’s 24-byte window and chunk metadata on every fragment. A record acknowledgement has a 51-byte header and 16-byte body; encoded control has a 61-byte header. Lower-layer framing contributes its own serialized airtime. These bytes consume resources while scientific payload measures exclude them. A shared half-duplex interface prevents simultaneous local transmit and receive service. Receive-state energy includes collided, corrupted and overheard waveforms, before application reception and duplicate checks.

An attempt finishes transmitting when local physical outcomes for its fragments are known. The acknowledgement grace starts from that event, rather than early queue admission. Local refusals settle the attempt and allow bounded retry. The reverse acknowledgement uses the actual forwarding path and can be lost or delayed. Receiver arrival and sender confirmation remain separate; duplicate data cannot create a second surface delivery. A candidate with r∈{0, 1, 3} retries permits r+1 attempts over an acoustic leg’s lifetime. A hard record deadline bounds lower-layer stalls. The hybrid MEDIUM bucket has one credit, replenished on the 1-s service tick at the candidate rate, preserving the discrete refill effect.

### Appendix A.1. Optical Contacts and Finite Custody

An acoustic wake carries a finite listening interval and receive credit. The gateway emitter is powered for admitted sessions, including startup and waiting. Optical DATA carries a 69-byte descriptor in addition to payload; its receipt is confirmed by a 28-byte acoustic control body. The sender retains its copy until confirmation. A bounded receipt cache supports duplicate acknowledgements while preserving unique custody. Nominal optical success is one inside the contact region; contact loss, power, admission and reverse-control failures remain possible. This is an optimistic timed optical service condition with explicit acquisition and confirmation costs.

Continuous contact is checked over the entire serialization interval. The start/end distances and the common speed bound give a conservative bound on intermediate separation through the triangle inequality. The test prevents endpoint-only acceptance when a vehicle could leave the 30 m region between those endpoints. Receiver admission is still an intermediate location; surface receipt defines end-to-end completion.

The archive, cable staging, persistent outbox and carry store are independent finite allocations. Each vertical link serves all priorities using ten wire bits per byte, 16-byte data/acknowledgement descriptors and an additional 24-byte LOW descriptor. After a modeled 10 ms durable gateway admission, the source waits for the returned cable acknowledgement before ending the admission transaction. Its acquisition backup remains in the finite archive under the retention rule. Cable retry is limited to three attempts per credit epoch, with finite source retention and explicit backpressure. Source backups remain physical copies, separate from the unique-record conservation count. Control reservations, optical receipt/session state and adaptive report storage are deducted from the vehicle allocation. The stated staging limits describe the modeled application buffers and their admission accounting.

### Appendix A.2. State Energy and Mission Envelope

Let $B_{i}$ be owner *i*’s initial battery energy and $P_{i}\left(t\right)$ its combined draw from the operating baseline, acoustic state and additional cable/optical loads. Its consumption is

(A2) $$E_{i}\left(t\right)=\min\;\left\{B_{i},\int_{0}^{t}P_{i}\left(u\right)\;du\right\}.$$

This integral definition gives the finite battery its operational meaning: depletion stops or rejects further service, and a waveform cannot deliver if its emitter or receiver loses power before completion. Over an interval of constant power before depletion, it reduces to the familiar state charge ΔE=PΔt; summing those charges implements the integral. The ledger partitions elapsed interface time and clips activity at the observation end. Overlapping receptions share one physical receive interval, preventing repeated charges of the same active receiver. Optical conversion loss, acquisition/logging and vertical ingress are charged at their respective owners independently of delivery success.

The 900 W vehicle baseline is an engineering value at the scale implied by the documented 67 kWh endurance; study interfaces draw additional energy from that same battery. Each proposed action includes offload, continuous return, external recovery and 20% reserve. The route construction uses 100 m horizontal turns, a 50 m minimum spatial radius, maximum pitch of 45 degrees and submerged service rendezvous. A full 100-meter-radius circle at 3 kn takes approximately 407.1 s, explaining the planner’s discretized service allowances. Calm water equates ground speed with prescribed water-relative speed. These checked geometric assumptions define the mission envelope alongside the published battery reference.

The artifact preserves complete parameters, generated inputs and owner/event ledgers. Component checks cover custody and duplicates, finite ingress, optical control, state energy, continuous routes and causal planning. Completed executions are independently audited against the offered source cohort, including incomplete windows and residual storage. These checks establish consistency of the implemented simulation instance; its performance retains the stated hardware assumptions and finite mission availability.
