# Supplementary material for: Direct Acoustic Versus AUV-Assisted Hybrid Reporting for Subsea Pipeline Monitoring: A Matched End-to-End Evaluation
Source: Sensors (Basel). 2026 Oct 8;26(19):6337. doi: 10.3390/s26196337 (PMC13667693; doi:10.3390/s26196337)
Supplement: Supplementary file 1 [file sensors-26-06337-s001.zip › sensors-4581665-supplementary.pdf]

Supplement S1: Preserved submitted-model analyses

Manuscript sensors-4581665

Direct Acoustic versus AUV-Assisted Hybrid Reporting for Subsea Pipeline Monitoring:
A Matched End-to-End Evaluation

Scope and original protocol

This supplement preserves the six results tables from the submitted manuscript, with their original entries, captions, uncertainty summaries and model boundary. They document the earlier experiment; they are not additional runs of the revised V6 model. Their retention does not imply that the revised and submitted estimates measure the same effect. In particular, delivered-record delay, record completion and timely recovery of a multi-chunk acquisition window are different outcomes.

The submitted reference used a 12.6-km corridor, eight gateways, fifteen surface stations and 100-record gateway storage. Aggregate arrivals were 0.05 record/s, with HIGH/MEDIUM/LOW proportions 0.10/0.30/0.60. Payloads were 64 bytes, 256 bytes and 10 or 25 kB, with deadlines of 120, 600 and 30,000 s. Finite experiments stopped generation at 4000 s and allowed queues to drain until 23,450 s. Continuous experiments kept generation active until 91,798.03 s and sampled five equivalent post-warm-up patrol boundaries. The submitted acoustic service used 31.2 kbit/s; optical service used 2.5 Mbit/s within a 30-m contact region. These are the historical scenario settings, not the revised hardware configuration.

The correspondence is submitted Table 4 → S1 (finite comparison), Table 5 → S2 (acoustic margin), Table 6 → S3 (fallback age), Table 7 → S4 (continuous service), Table 8 → S5 (cycle-end storage), and Table 9 → S6 (idle power). No original table entry has been recalculated or replaced.

Finite traffic and the scheduling control

The original direct-FIFO control changes service order; direct priority changes that order while retaining the acoustic path. Hybrid priority additionally changes the routine-record path. This decomposition remains useful within its original model. The FIFO control in the revised campaign instead changes gateway *eviction*, with priority service unchanged; the two controls answer different questions. Delay entries below condition on delivered records, and the energy entries retain the restricted accounting explained on the final page of this supplement. RDR counts completely received surface records divided by generated records. Acoustic bytes include headers, hops and retries. Normalized acoustic attempt load sums transmission time across senders and divides by the observation horizon; concurrent spatial links contribute independently.

Table S1: Finite-traffic reference results for 25-kB routine records at $\lambda = 0.05$ records/s, followed by queue drain and averaged over 20 common seeds. The primary effect is hybrid priority minus direct priority; percentage effects are percentage points.

Metric	Direct FIFO	Direct priority	Hybrid priority	Primary effect [95% CI]
Completion and deadline performance				
End-to-end RDR (%)	99.80	99.80	98.59	−1.22 [−1.96, −0.47]
HIGH deadline misses (%)	1.18	0.24	0.22	−0.02 [−0.71, 0.67]
MEDIUM deadline misses (%)	0.43	0.58	4.67	+4.09 [1.67, 6.50]
Delivered-record delay				
HIGH conditional delay (s)	20.7	14.4	**3.45**	−10.99 [−13.93, −8.06]
MEDIUM conditional delay (s)	19.1	13.9	233.8	+219.9 [201.1, 238.8]
LOW conditional delay (s)	33.7	34.7	5924	+5890 [5640, 6139]
Acoustic demand				
Acoustic transmitted bytes (MB)	4.83	4.82	**4.28**	−0.54 [−0.64, −0.44]
Normalized acoustic attempt load (%)	5.28	5.27	4.68	−0.59 [−0.71, −0.48]
Deployment energy accounts				
Submerged-device energy (kJ)	11.99	11.99	**2.40**	−9.59 [−10.13, −9.05]
Incremental-patrol energy (kJ)	12.90	12.89	11.95	−0.94 [−1.16, −0.72]
Dedicated-mission energy (MJ)	0.0129	0.0129	5.324	+5.311 (descriptive)

Acoustic-margin and fallback-age sensitivities

The margin study compares direct and hybrid priority using ten common seeds at each synthetic acoustic margin. Its numerical reference differs from the twenty-seed sample in Table S1. The margin is an input to the historical reception model, not a measured channel calibration. This experiment has not been rerun as a V6 margin sweep.

Table S2: Sensitivity to the synthetic acoustic margin. D/H denotes direct priority/hybrid priority; Δ is hybrid minus direct. Values use 10 matched seeds at each margin.

M (dB)	Direct RDR (%)	Hybrid RDR (%)	ΔRDR [95% CI]	HIGH delay D/H (s)	Acoustic D/H (MB)
52	99.81	99.24	−0.57 [−1.35, 0.20]	15.08 / **3.56**	4.95 / 4.35
58	99.85	99.24	−0.62 [−1.62, 0.39]	4.82 / **3.30**	3.21 / 3.20
64	99.85	99.24	−0.62 [−1.62, 0.39]	4.82 / **3.30**	3.21 / 3.20

The fallback-age study changes the waiting threshold while retaining the historical one-credit bucket and its 0.015-record/s refill. Thus, the 60-s setting is the best age among those tested under finite traffic, not a global optimum or a test of increased refill capacity. Reviewer R1.3 motivates the separate 0.020/0.030-record/s controls in the revised campaign. Pooled counts below audit the events; they do not replace independent runs as replication units.

Table S3: Hybrid sensitivity to the MEDIUM fallback age gate. Intervals are Student 95% intervals over 20 independent runs; pooled ratios provide an event-level audit of the run-level inference.

τ_M (s)	RDR (%)	MEDIUM miss (%) [95% CI]	Pooled miss (%)	MEDIUM delay (s)	MEDIUM expirations
60	**98.74**	**4.17** [1.83, 6.51]	**4.62**	**178.6**	51
120	98.59	4.67 [2.18, 7.16]	5.17	233.8	57
240	97.85	7.14 [3.38, 10.89]	7.89	341.3	87

Continuous production under the submitted patrol

The following continuous experiment uses the repeated historical patrol and two independent record sizes. Its status misses characterize the stated policy and refill setting. They do not establish an intrinsic limitation of hybrid architectures. The revised study instead includes finite acquisition, byte-limited storage, acknowledged custody and one finite vehicle mission; its complete-window percentages cannot be substituted into this table.

Table S4: Continuous-arrival service over five complete post-warm-up patrol cycles. Metric entries are run means with 95% Student half-widths over five runs. A/D/S/P are pooled terminal seabed-access losses, deadline expirations, stored records, and pending transactions; every record passed admission.

Service	LOW (kB)	Terminal RDR (%)	Mature HIGH miss (%)	Mature MEDIUM miss (%)	Pooled A/D/S/P
Direct priority	10	99.983 ± 0.012	0.000 ± 0.000	0.015 ± 0.041	1/0/0/3
Direct priority	25	99.856 ± 0.037	0.360 ± 0.268	0.309 ± 0.132	29/0/1/3
Hybrid priority	10	91.459 ± 0.790	0.452 ± 0.685	15.015 ± 3.100	0/844/1113/0
Hybrid priority	25	91.459 ± 0.807	0.539 ± 0.669	15.279 ± 3.317	0/844/1113/0

Cycle-end storage and the limits of terminal diagnosis

The five storage observations are taken at 21,559.606, 39,119.212, 56,678.818, 74,238.424 and 91,798.030 s. Sampling equivalent patrol phases avoids confusing within-cycle queue oscillations with accumulation. The ordinary-least-squares trends describe only these five observed cycles. A stable queue can coexist with missed deadlines because expiration removes work. An empty AUV queue at a cycle end does not establish that offload capacity was sufficient throughout the cycle. The revised stage analysis addresses this causal distinction raised by R1.6 and R2.4.

Table S5: Mean stored gateway-plus-AUV records at exact patrol-cycle ends. Trends are run means with 95% Student half-widths over five runs and use the five displayed cycle-end points.

Service	LOW (kB)	Cycle 1	Cycle 2	Cycle 3	Cycle 4	Cycle 5	Trend (records/cycle)
Direct priority	10	0.0	0.2	0.0	0.0	0.0	-0.02 ± 0.06
Direct priority	25	0.2	0.6	0.8	0.6	0.2	0.00 ± 0.23
Hybrid priority	10	223.0	215.8	224.0	218.6	222.6	0.20 ± 10.19
Hybrid priority	25	223.0	215.8	224.2	218.8	222.6	0.22 ± 10.14

Historical energy boundary and idle-power sensitivity

Table S6 varies the historical idle allowance while retaining the modeled active-energy difference. It illustrates why a local percentage reduction depends on the accounting denominator. The allowance is assigned per nominal submerged position and redistributed across communication endpoints; it is not measured consumption of the revised logger/modem assembly.

Table S6: Sensitivity of submerged-device energy to idle or hibernation power per nominal submerged position. Values compare hybrid priority with direct priority; 0.5 mW is the low-power reference input.

Idle/hibernation power per position	Direct priority (kJ)	Hybrid (kJ)	Reduction (%)
0.1 mW	10.30	0.71	93.1
0.5 mW	11.99	2.40	80.0
1 mW	14.10	4.51	68.0
2 mW	18.32	8.73	52.3
5 mW	30.98	21.39	31.0
10 mW	52.09	42.50	18.4
20 mW	94.30	84.71	10.2
50 mW	220.93	211.34	4.3

The submitted event ledger charged optical energy on successful transfers and acoustic receive energy after successful reception. Its reverse acknowledgement was an idealized immediate success with an energy charge, not a transmitted reverse packet exposed to propagation, contention and loss. Consequently, it does not account completely for failed or partial activity, listening and acknowledged custody. The dedicated-mission term used a declared constant-plus-cubic vehicle scenario, without establishing a calibrated HUGIN energy/mission model. These qualifications apply to the energy rows in Table S1 as well as Table S6.

The incremental-patrol account assumes that collection leaves an independently commissioned patrol unchanged. Its numerical saving is not a demonstrated deployment benefit when collection adds vehicle time, waiting or detours. The revised manuscript uses explicit finite-owner state energy and reports total system consumption together with complete timely observations. Neither the old energy percentages nor a before/after ratio across model versions quantifies a hardware efficiency improvement.

These preserved analyses remain records of their declared simulation protocol. Their replacement in the active revised comparison does not establish that every original estimate was mathematically erroneous. No new experiment or physical validation is presented in this supplement; the current manuscript and its versioned evidence package define the revised claims.
